# Early Insight Into the Retrospective Data of a Case Series on Type 2 Diabetes Mellitus on Alternate-Day Dosing of Oral Semaglutide: Utopia or Reality?

**DOI:** 10.7759/cureus.37065

**Published:** 2023-04-03

**Authors:** Soumyabrata RoyChaudhuri, Anirban Majumder, Debmalya Sanyal, Soma Chakraborty, Susama Chuyan

**Affiliations:** 1 Endocrinology, Kali Prasad Chowdhury Medical College & Hospital, Kolkata, IND; 2 Nutrition, Diabetes-Obesity-Thyroid-Hormone Clinic, Kolkata, IND; 3 Diabetes Educator, Adopt Endocrine Diabetes Care, Kolkata, IND

**Keywords:** type 2 diabetes mellitus, oral semaglutide, glycaemic parameters, ambulatory glucose profile (agp), alternate day dosing

## Abstract

Introduction

Oral semaglutide, with a long half-life of seven days, is the first oral-based peptide drug and is used as an antidiabetic for the reduction of glycosylated hemoglobin (HbA1c). Oral semaglutide, like other glucagon-like peptide-1 receptor agonists (GLP1RAs), is costly and has gastrointestinal (GI) side effects, especially with a 14 mg dose. In the real world, some type 2 diabetes mellitus (T2DM) patients on 14 mg oral dose adopt an alternate-day strategy to minimize unwanted GI symptoms. In this study, we analyzed the ambulatory glucose profile (AGP) data of patients with T2DM who were on 14 mg alternate-day oral semaglutide therapy.

Methods

This retrospective observational study evaluated the AGP data of 10 patients on alternate-day dosing of 14 mg oral semaglutide. The AGP data over a period of 14 days on a single group of patients were analyzed without any control group or randomization and are presented as a case series. AGP monitoring, using Freestyle Libre Pro (Abbott, Illinois, United States), is a standard operating procedure of the endocrinology department for all T2DM patients who were put on oral semaglutide therapy. The AGP data of the glycemic parameters time-in-range (TIR), time-above-range (TAR), and time-below-range (TBR), were compared between the days when oral semaglutide was consumed (days-on-drug) versus the days when oral semaglutide was not consumed (days-off-drug). The statistical analysis was done with Statistical Package for Social Sciences (SPSS) version 21.0 (IBM Corp., Armonk, NY).

Results

We applied the Shapiro-Wilk test (sample size <50) for normality testing; the TIR values of days-on-drug and days-off-drug showed high p values (p =0.285 and 0.109), respectively. This indicated that TIR values days-on-drug and days-off-drug were normally distributed. Although, the distribution of TAR and TBR values days-on-drug and days-off-drug, were not normal as they had small p values (p< 0.05). Hence, further analysis of the paired set of data was done using the Wilcoxon signed-rank test. It revealed no difference in TIR, TAR, and TBR between the two groups (days-on-drug and days-off-drug).

Conclusion

Throughout the period of observation, the glycemic metrics (TIR, TAR, and TBR) remained steady with a 14 mg alternate-day oral semaglutide regimen.

## Introduction

A significant percentage of insulin secretion in a healthy person in response to a meal is mediated by the incretin hormones, glucagon-like-peptide-1 (GLP-1) and glucose-dependent-peptide (GIP) [1]. GLP1 is the principal hormone responsible for the insulinotropic response after the meal [2]. Since the discovery of GLP1, which has an ultra-short half-life of minutes, there was a keen interest to prepare synthetic analogs with longer half-lives and put them into therapeutic use. Exenatide, a short-acting glucagon-like peptide-1 receptor agonist (GLP1RA), built on the exendin backbone, was the first GLP1RA to receive approval for therapeutic use in people with type 2 diabetes mellitus (T2DM) from the United States Food and Drug Administration (USFDA) in 2005 and from the European Medicines Agency (EMA) in 2006. Later, six other agents belonging to the same class were developed, namely, short-acting lixisenatide, intermediate-acting liraglutide, and long-acting once-weekly (QW) - semaglutide, albiglutide, dulaglutide, and extended-release exenatide. Although the dosing schedules and pharmacokinetics of the GLP1RAs differed from one another, all of them needed administration through the subcutaneous route as injections. GLP1RAs, by correcting multiple well-recognized pathophysiological defects of T2DM, led to a moderate reduction of glycated haemoglobin (HbA1c), body weight, and systolic blood pressure with a low risk of hypoglycemia [3,4]. GLP1RAs are recommended as the first-line injectable therapy, ahead of insulin, in people with T2DM, who fail to achieve control on multiple oral antidiabetic agents [5]. Cardiovascular outcome trials (CVOTs), namely, LEADER, SUSTAIN-6, HARMONY, and REWIND, established the role of GLP1RAs in the reduction of major adverse cardiovascular events (MACE) [6-9]. These outcomes prompted the European Society of Cardiology (ESC) to recommend GLP1RAs as the first line of therapy in people with established atherosclerotic cardiovascular disease (eASCVD) in 2019 [10]. However, despite the positioning and availability of data regarding their exclusive metabolic and cardiovascular benefits, there has been a poor global acceptance of the molecule and inertia on the part of healthcare professionals to prescribe the injectable GLP1RAs [11,12]. In a large US-based survey, gastrointestinal (GI) side effects and the injectable route of administration appear to be the prime hurdles for GLP1RAs initiation and the prime reason for their premature discontinuation [13]. GI side effects are typical of the GLP1RA class of drugs due to their inherent mechanism of action but are manageable using a go-slow-go-low approach [14,15]. However, an oral formulation appears to be the best option to improve acceptance and adherence to GLP1RA therapy by mitigating the fears of injection.

Semaglutide, a GLP1RA, is the first oral-based peptide drug and has a long half-life of seven days. Semaglutide is co-formulated with an absorption enhancer, namely, salcaprozate sodium or sodium N-(8-[2-hydroxy benzoyl]amino) caprylate (SNAC) [16]. SNAC provides a transient local increase in pH that helps protect semaglutide against proteolytic degradation in the stomach and facilitates drug absorption via the transcellular route across the gastric epithelium in a concentration-dependent manner [16-18]. The Peptide InnOvatioN for Early diabEtes tReatment (PIONEER) program showed that oral semaglutide caused a dose-dependent reduction of HbA1c [19], and it was approved by the USFDA in 2019. One percent of oral semaglutide is absorbed from the stomach and the rest is degraded in the gastrointestinal transit. A steady state exposure is achieved following four to five weeks of oral administration. It is to be noted that once the oral semaglutide is absorbed, its pharmacokinetic and pharmacodynamics is the same as that of QW subcutaneous semaglutide, with a similar long half-life of seven days [18,20,21].

Oral semaglutide, like other GLP1 RAs, exhibited GI side effects across the PIONEER trials, of which nausea was the most common followed by vomiting, dyspepsia, diarrhea, and constipation. However, incidences of adverse GI events in the PIONEER trials match the pooled data of liraglutide therapy among Indians [22]. The GI side effects of oral semaglutide in the PIONEER 4 trial were observed at around eight weeks. It is much later than the GI side effects of liraglutide observed at around two weeks. This can be attributed to the different escalation regimens of the two drugs - monthly for oral semaglutide versus weekly for liraglutide. The onset of GI symptoms in the PIONEER 4 trial with oral semaglutide establishes that the GI symptoms are majorly seen at the 14 mg dose. Also in real-world usage, in line with the PIONEER trials, GI symptoms seem to appear in most people with T2DM at the 14 mg dose. Some of the patients with T2DM on 14 mg oral semaglutide came back for a follow-up in our department, adopting by themselves a protective strategy of alternate-day usage of the drug, to minimize the adverse GI symptoms. When we looked at the self-monitoring of blood glucose (SMBG) chart of these patients, most of the SMBG values with oral semaglutide (days-on-drug) versus without oral semaglutide (days-off-drug) seemed strikingly similar. In this study, we analyzed the ambulatory glucose profile (AGP) of patients with T2DM following a self-adopted alternate-day dosing schedule of 14 mg oral semaglutide.

## Materials and methods

This retrospective observational study evaluated the AGP of 10 patients with T2DM who were on alternate-day dosing of 14 mg oral semaglutide (adopted as a self-devised protective schedule to minimize GI adverse events) visiting the endocrine outpatient department (OPD) of a tertiary care hospital of Eastern India. SMBG with a personal glucometer was routinely advised as a standard of care for all patients on oral semaglutide. AGP, using the Freestyle Libre Pro glucose monitoring system from Abbott (Illinois, United States), was also offered for two weeks as a standard of care to all patients on oral semaglutide after at least four weeks of 14 mg oral semaglutide usage. The study was intended to look at any differences in the time-in-range (TIR), time-above-range (TAR), and time-below-range (TBR), on the AGP data of the patients, days-on-drug versus days-off-drug. 

Inclusion criteria

These criteria included (1) age from 18 to 70 years; (2) T2DM diagnosed based on the American Diabetes Association criteria; (3) receiving self-designed alternate-day 14 mg dose of oral semaglutide along with other unaltered oral antidiabetic drugs (OADs) for a period of at least six weeks (four weeks of alternate-day oral semaglutide plus two weeks of AGP monitoring on the same schedule); (4) completed the two weeks of AGP monitoring at least after four weeks of 14 mg alternate-day oral semaglutide administration; (5) could reliably recollect the days-on-drug and the days-off-drug during the AGP monitoring period.

Exclusion criteria

These criteria included (1) receiving 14 mg oral semaglutide daily or irregularly; (2) receiving 14 mg oral semaglutide on the alternate-day schedule of fewer than four weeks; (3) change in OADs during the AGP monitoring period; (4) incomplete AGP monitoring; (5) suffered from an intercurrent illness during the 14 days of AGP monitoring or underwent hospitalization.

The Statistical Package for Social Sciences (SPSS) version 21.0 (IBM Corp., Armonk, NY) was used for statistical analysis.

Ethical considerations

The eligible patients had discussed the risk of taking oral semaglutide drug (potential adverse effects) and its therapeutic benefit. Written informed consent was obtained from every patient as a standard operating procedure of the department. Ethics committee approval was not sought because the study was retrospective and observational. The Institutional Ethics Committee as a matter of principle waives off the requirement of permission for investigator-initiated retrospective data analysis-driven projects. Confidentiality and anonymity were meticulously maintained. All the tenets of the Helsinki declaration relating to bioethics policy were strictly adhered to.

## Results

All analyses were performed using the statistical tools of SPSS ver 21.0. AGP data were analyzed from the cohort of 10 patients with T2DM who fulfilled the inclusion and exclusion criteria. The mean age of the cohort was 58.3 ± 11.26 years, with four male and six female patients (a ratio of 2:3), and the mean BMI was 30.3 ±-4.03 kg/m^2^ (Table 1).

**Table 1:** Demography and AGP AGP: ambulatory glucose profile

Serial no	Age	Sex	BMI	Days-on-drug			Days-off-drug		
--	--	--	--	TIR 1	TAR 1	TBR 1	TIR 2	TAR 2	TBR 2
1	57	F	34	85	12	2	86	12.8	0.57
2	55	F	26	85	0	14	81	0	18
3	55	M	26	92	3.4	4.1	94.2	1.2	4.4
4	50	F	34	99	0	0.8	99	0	0.5
5	64	F	38	71.5	4	24.4	76.5	6.1	17.2
6	48	M	30	61	37.6	1.4	74	24	1.4
7	59	F	31	62.7	0	37.2	74.2	0	25.7
8	49	M	27	83	17	0	79.4	19.2	1.14
9	59	M	27	65.5	0.71	33.7	71.8	0.5	27.5
10	87	F	30	99.4	0.14	0.4	99.1	0.85	0
Mean value	58.3	4	30.3	80.41	7.48	11.8	83.52	6.46	9.64
Standard deviation	11.26	6	4.03	14.46	12.09	14.69	10.49	9.01	11.21
Median	56		30	84	2.05	3.05	80.2	1.02	2.9
IQR	7.75		6.25	23.25	9.96	20.85	17.37	11	17.08

By applying the Shapiro-Wilk test for normality testing (for sample size <50), it was found the paired TAR and TBR data are non-normally distributed, although TIR data were found normally distributed. As the data were paired with non-normal distribution, it was decided to apply a non-parametric Wilcoxon signed rank test to find out the significant difference in the paired sample at two different time points (Tables 2-4).

**Table 2:** Non-parametric tests: descriptive statistics TIR: time-in-range; TAR: time-above-range; TBR: time-below-range

Days	Glycemic Metrics	N	Mean	Std. Deviation	Minimum	Maximum	Percentiles		
							25th	50th (Median)	75th
Days-on-drug	TIR1	10	80.4100	14.45863	61.00	99.40	64.8000	84.0000	93.7500
	TAR1	10	7.4850	12.08577	.00	37.60	.0000	2.0550	13.2500
	TBR1	10	11.8000	14.68960	.00	37.20	.7000	3.0500	26.7250
Days-off-drug	TIR2	10	83.5200	10.48828	71.80	99.10	74.1500	80.2000	95.4000
	TAR2	10	6.4650	9.00704	.00	24.00	.0000	1.0250	14.4000
	TBR2	10	9.6410	11.20657	.00	27.50	.5525	2.9000	19.9250

**Table 3:** Wilcoxon signed-rank test a. TIR2 < TIR1, b. TIR2 > TIR1, c. TIR2 = TIR1, d. TAR2 < TAR1, e. TAR2 > TAR1, f. TAR2 = TAR1, g. TBR2 < TBR1, h. TBR2 > TBR1, i. TBR2 = TBR1 TIR: time-in-range; TAR: time-above-range; TBR: time-below-range

-----	N	Mean Rank	Sum of Ranks
Negative Ranks	3^a^	3.33	10.00
Positive Ranks	6^b^	5.83	35.00
TIR2 - TIR1			
Ties	1^c^		
Total	10		
Negative Ranks	3^d^	4.50	13.50
Positive Ranks	4^e^	3.63	14.50
TAR2 - TAR1			
Ties	3^f^		
Total	10		
Negative Ranks	6^g^	5.58	33.50
Positive Ranks	3^h^	3.83	11.50
TBR2 - TBR1			
Ties	1^i^		
Total	10		

**Table 4:** Test statistics a. Wilcoxon signed-ranks test; b. Based on negative ranks; c. Based on positive ranks

Test Statistics ^a^			
	TIR2 - TIR1	TAR2 - TAR1	TBR2 - TBR1
Z	-1.481^b^	-.085^b^	-1.304^c^
Asymp. Sig. (2-tailed)	.139	.933	.192

A Wilcoxon signed-rank test showed that TIR, TAR, and TBR values days-on-drug vis-a-vis days-off-drug did not have a statistically significant difference. The change in TIR values between the two groups was Z = -1.481 (p = 0.139). The changes in TAR and TBR values days-on-drug and days-off-drug were Z = -0.85 (p = 0.933) and Z=-1.304 (p=0.192), respectively (Table 4). Also, the median TIR1 and TIR2 values of the days-on-drug (84 and 3.05) and days-off-drug (80.2 and 2.9) were almost similar (Table 1).

## Discussion

It has always been a challenge for healthcare professionals to ensure that patients adhere to the prescribed medications for chronic disorders like T2DM. One of the prime reasons for non-adherence is the rising cost of medication, which in turn can lead to adverse clinical outcomes [23,24]. Alternate-day dosing of drugs with a long half-life has always been an interesting point of clinical and health economics research. In this regard, atorvastatin, with a half-life of 14 hours, and rosuvastatin, with a longer half-life of 19 hours, were demonstrated as safe and effective therapeutic options in lowering total cholesterol with alternate-day therapy [25,26].

With the availability of long-acting OADs, attempts have been made to try and administer the drugs on an alternate-day basis without compromising their efficacy but reducing the cost. Linagliptin, a dipeptidyl peptidase 4 (DPP4) inhibitor, with a long elimination half-life of about 130 hours and a high volume of distribution, has long-lasting DPP4 inhibitory effects in vivo. There is no significant difference in fasting plasma glucose (FPG), postprandial plasma glucose (PPPG), and HbA1c between alternate-day and daily linagliptin therapy [27]. In one subject within the cohort, continuous glucose monitoring (using CGMS iPro2; Medtronic Minimed, Northridge, CA, USA) didn’t show any significant difference in the 24-hour blood glucose profile (both during day and night) between the days of linagliptin administration and the days without linagliptin administration. However, there were no consensus statements or guidelines at the time of publication of the article defining the ranges of TIR as a metric of glycaemic control.

In 2017, TIR was recommended as a short-term measure of glycemic control in both type 1 diabetes mellitus (T1DM) and T2DM by the International Consensus on the Use of CGMS. TIR has emerged as an intuitive metric that denoted the amount of time as a percentage of the 24-hour period for which the person’s glucose level remains within the proposed target range, which for T2DM is 70-180 mg/dl and for pregnancy is 63-140 mg/dl [28,29].

HbA1c analysis reflects the average glucose control over the last three months but is influenced by various other factors apart from glucose concentration and fails to represent accurate glycemic status under many circumstances [30]. It also fails to indicate glycemic variability (GV), viz. the daily crests and troughs of glucose levels, which have positively been implicated in the development of micro and macrovascular complications of diabetes [31]. The five landmark trials - United Kingdom Prospective Diabetes Study (UKPDS), PROspective pioglitAzone Clinical Trial In macroVascular Events (PROactive), Action to Control Cardiovascular Risk in Diabetes (ACCORD), Action in Diabetes and Vascular Disease: Preterax and Diamicron Modified Release Controlled Evaluation (ADVANCE), and Veterans Affairs Diabetes Trial (VADT), demonstrated that intensive HbA1c lowering did not reduce non-fatal stroke, cardiovascular mortality, and all-cause mortality [32]. This observation further challenged the credibility of using HbA1c as the principal and sole therapeutic target for glucose control. In recent years, the focus is shifting from HbA1c alone to HbA1c plus TIR as a measure of glycaemic control.

There have been ongoing efforts by researchers to try and establish a relationship between TIR and HbA1c. It is estimated that for every 10% change in TIR, there is a corresponding 0.8% change in HbA1c in a mixed population of T1DM and T2DM [33]. In T2DM, a mean TIR of 84% corresponds with an HbA1c of 7 % [34]. It is also observed that a mean TIR of > 70% in T2DM of Asian Indians relates to an HbA1c level of <7.5% [35]. The mathematical correlation of HbA1c for a given TIR based on data derived from these studies is given in Table 5.

**Table 5:** Correlation between TIR (70–180 mg/dL) and HbA1c as estimated by studies TIR: time-in-range; HbA1c: glycated hemoglobin

Authors	Type of population	Correlation coefficient (r) between TIR and HbA1c
Vigersky and McMahon [33]	Mixed T1DM / T2DM (n = 1,137)	−0.84
Dixon FR et al [34]	T2DM (n = 194)	−0.78

There is no statistically significant difference between the average TIR1 (80.41±14.46 %) days-on-drug compared to the average TIR2 (83.52±10.49 %) days-off-drug in our cohort (Table 1). If we extrapolate these findings to the Asian Indian-specific data generated by Kesavadev et al. [35], the HbA1c of both sets of TIR is comparable and well below 7.5%. TBR, suggestive of the time spent in hypoglycemia, is also analyzed. There is no statistically significant difference between the average TBR1 (11.8±14.69 %) days-on-drug and the average TBR2 (9.641±11.21%) days-off-drug (Table 1). A Wilcoxon signed ranks test was conducted on these two sets of values to find any statistically significant difference between days-on-drug versus the days-off-drug (Table 3 and Table 4). This small set of data indicates that after attainment of the steady state (in four weeks) on alternate-day oral semaglutide, the matrices of glycaemic control (TIR, TAR, and TBR) remain unchanged and unaltered throughout the period of observation in the next two weeks. There was no documented symptomatic hypoglycemia observed as per the SMBG charts of the cohort during the period of AGP monitoring. Therefore, semaglutide, with a long half-life of seven days, can possibly be administered as an alternate day regimen, leading to a major reduction in the cost of monthly medication, the prime cause of GLP1RA discontinuation, resulting in adherence to the therapy.

Limitations

The duration of alternate-day intake of oral semaglutide in this study is short. We need to carry out long-term studies, involving a larger number of patients/participants with alternate-day dosing of oral semaglutide so that the impact of the dosing schedule on HbA1c (weighted mean of three months) as well as on TIR, TAR, and TBR, can be assessed along with a comparison of FBS and PPBS values. The design of further studies should also have a control arm with age and sex-matched patients/participants on daily dosing of oral semaglutide. We did not do a retinopathy evaluation for this subset of patients. Focused attention on the extra glycaemic benefits like weight loss, systolic blood pressure reduction, and impact on eye, renal, and cardiovascular outcomes need to be given before we can propose alternate-day dosing of oral semaglutide as a therapeutic option.

## Conclusions

Oral semaglutide is a novel drug containing a peptide in an oral pill. This preliminary, retrospective observational case series of patients with T2DM on a self-prescribed alternate-day regimen of oral semaglutide shows steady glycemic metrics (TIR, TAR, and TBR) throughout the period of observation. Long-term evidence combining AGP with other conventional measures of glycemic control, viz. FPG, PPPG, and HbA1c, along with convincing data regarding weight benefit, cardiovascular benefit, and renal benefit need to be on board for alternate-day dosing of oral semaglutide to be an accepted therapeutic modality.
